# Stakeholder views on addressing challenges to the implementation of social prescribing in the United Kingdom

**DOI:** 10.3389/frhs.2024.1413711

**Published:** 2024-10-11

**Authors:** Sima Rafiei, Mahsa Honary, Barbara Mezes

**Affiliations:** ^1^ School of Management Sciences, Lancaster University, Lancaster, United Kingdom; ^2^ Department of Primary Care and Mental Health, Institute of Population Health, University of Liverpool, Liverpool, United Kingdom

**Keywords:** implementation challenges, social prescribing, stakeholder, qualitative study, participatory method

## Abstract

**Objectives:**

This study aimed to understand the key barriers to successfully implementing Social Prescribing (SP) initiatives from different perspectives.

**Methods:**

An in-depth process evaluation using a multi-method qualitative design was conducted. Qualitative data was collected via semi-structured interviews (*N* = 23) and Focus Group Discussion (FGD’ *N* = 4). Twenty-three stakeholders took part in the study, including community support providers (*n* = 7), SP link workers (*n* = 6), service users (*n* = 6), NHS employees/referrals, and those who were involved in SP leadership and coordination (*n* = 4). MAXQDA Version 20.0 was used for management and data analysis.

**Results:**

We identified eight themes representing challenges for a successful implementation of a SP programme. The themes included (i) financial issues and sustainability, (ii) human resources challenges, (iii) partnership working challenges, (iv) inadequate and inconsistent implementation, (v) information system challenges, (vi) referral system issues, (vii) training and knowledge gaps, and (viii) accessibility and privacy concerns.

**Conclusion:**

Study findings provide insight for commissioners, providers, and link workers to guide the delivery of appropriate SP services by identifying a range of factors that hinder the successful implementation of the programme. Future policy, service development, and research should consider tackling these challenges and generating different ideas for potential solutions to address the root causes of problems.

## Introduction

People's health and well-being are not only affected by medical factors; rather some significant non-medical determinants contribute to over 80% of health consequences (1). Mental health problems along with negative Social Determinants of Health (SDH) such as poverty, unemployment, housing issues, social isolation, domestic violence, poor access to education, grief, and loss can have a more significant impact on people's health status (1, 2). In addition to the substantial effects that such aspects have on the health of society, they impose additional burdens on health systems and health providers, mainly on primary care (3). Therefore, to effectively respond to people's non-medical needs, it is essential to provide them with adequate and high-quality health and social care in the community, a key aim of the Social Prescribing (SP) programme (4).

As a broad definition, SP is an approach that “makes the connection between people, and a range of community wellbeing activities which enables a variety of healthcare stakeholders to refer people to social services to empower them in achieving good health and wellness” (5). It is conceived that SP services can potentially empower healthcare providers to meet a more comprehensive range of patient needs, relieve the heavy burden on primary care services, deliver better patient-centered care, and improve patient outcomes. It is also believed that SP models expand the outdated boundaries of primary care by employing link workers who help connect patients with community groups and organisations for practical, social, and emotional support services (6, 7).

### Rational and theoretical framework

While promising results indicate the potential benefits of SP and community-based support, there are key challenges in the implementation of such complex interventions due to the heterogeneity of contexts and activities included (6). Accordingly, a recent report indicated a significant increase in the need for social prescribing while it also highlighted some barriers to accessing/taking up the offered support, which requires further exploration. Potential barriers may further increase health inequalities, which is especially concerning (7).

Based on a realist review, successful evaluation strategies should consider SP as a system of interacting pathways and components rather than one intervention (6). The authors recommended that evaluating such complex systems should include evidence to inform elements of the user pathway and consideration of contextual factors. Therefore, there is a need to understand the process of such a large government scheme; where different stakeholders comprise service users, community and NHS service providers, and SP link workers are included (8). This approach would help to better understand the referral process for community-focused activities in the current SP care infrastructure by identifying existing challenges.

### The importance of process evaluation

Process evaluation was our approach as it enabled us to gain an in-depth understanding of the key challenges perceived and experienced by different stakeholders during the implementation of SP programmes (9). Moore et al. (2014) defined process evaluation as “a study that evaluates the implementation, and contextual factors of a programme to identify how a system functions to achieve a certain set of goals” (10). Indeed, a well-conducted process evaluation is significant as it considers a broad range of implementation aspects including those that obstruct and enable the delivery of SP services that affect the program's outcome (10). Therefore, local authority commissioners and healthcare providers need to obtain realistic information about the challenges regarding the current SP service delivery system to inform future planning efforts and enhance service delivery and governance (11).

### Study objective

In this study, we aimed to better understand the referral/acceptance process for community-focused activities in the current SP care infrastructure and identify existing challenges to the implementation of the SP programme from different stakeholder perspectives.

## Methods

### Study design

A multi-method qualitative study was conducted to gain a comprehensive understanding of challenges that impede the successful implementation of the social prescribing program in the Lancashire and South Cumbria area, of the North West of the UK. Twenty-three online interviews and four semi-structured focus group discussions were conducted to collect information from stakeholders who directly took part in SP interventions.

### Participants

The inclusion criteria included (i) having experience of the social prescribing provision in Lancashire and South Cumbria, UK (ii) minimum age of 18 years old; (iii) willingness to participate in the study and provide written informed consent. Participants included community-based support providers from the Voluntary, Community, Social Enterprise (VCSE) sector (CP), SP Link Workers (LWs), Experts by Experience^1^/service users) (EbE), NHS employees and those who were involved in SP leadership and coordination (NHS). Interviewees included six experts by experience, six LWs, four health and social care professionals, and seven Community Providers (CPs). The recruitment process is shown in Supplementary Materials S1.

### Materials

To inform the interview and focus group topic guides (see Supplementary Materials S2), a scoping review of the literature was conducted to gain a preliminary understanding of the underlying problems and success stories in SP service delivery. The interview questions explored stakeholders’ perspectives of SP services, including potential challenges in SP, experiences of the SP referral process, perceived barriers to accessing support through SP, and their views on potential areas for improvement in how the SP system operates.

### Procedure

Participants were recruited through: (i) posters/flyers displayed in GP surgeries, public places, and community centers, such as libraries, and sites where SP activities were taking place, e.g., community hubs, and; (ii) through the networks of community project partners and the local Clinical Research Networks (CRN) and (iii) snowball sampling All potential participants received information about the study via email. Informed consent was obtained remotely and all participants completed the demographic questionnaire before take part in remote interviews. Semi-structured remote interviews (via Microsoft Teams) were undertaken by an experienced qualitative researcher with a total of 23 stakeholders in SP between June and July 2023. Online interviews typically lasted for about 45 min and all interviews were digitally recorded and transcribed verbatim for analysis. Following the interviews, 4 in-person focus groups were held- one group per participant group (i.e., LW, CP, EbE, NHS).

During the subsequent phase of data collection, four focus group discussions were conducted, each involving a distinct group of stakeholders. As the optimal size of the focus group is about six to eight participants and because a potential power of imbalance within the group might adversely affect the study objectives, researchers assigned an appropriate number of participants belonging to a single, analogous group of stakeholders for each of the discussion sessions (12, 13). To collect data, face-to-face focus groups were conducted by two researchers; one acted as a facilitator to facilitate group discussions and another attended the meetings as an observer to monitor group dynamics (14). Focus groups were held in August 2023 and each took approximately 2 h. The reason behind combining individual interviews with focus groups was to enhance our insight into both individual's and groups’ viewpoints, experiences, and perspectives which can help to inform developments in the SP system (15). Participants were compensated for their time.

Data included audio recording of the interviews and focus groups, and observation notes during the focus groups. The discussions were transcribed in full by the administrative staff member and consequently were entered into the MAXQDA software version 2020.

### Data analysis

Thematic analysis was used as a qualitative analysis technique, based on open coding accompanied by more detailed coding. To do so, SR read transcripts, refined, and developed the coding framework. In the next step, the research team discussed the coding framework, compared coding to measure the degree of agreement across and within the transcripts, and finalized a complete analytical picture of the data. The coding framework broadly covered themes on the subject of stakeholders’ viewpoints around the challenges faced in the implementation of SP. This process included the steps set out by Braun and Clarke: data familiarisation, development of initial codes, searching for themes, defining themes, and producing the report (16).

### Ethical considerations

Throughout all phases of the project, we adhered to the ethical standards set out in Lancaster University's Code of Practice and by research ethics guidelines specified by the research councils. In addition, ethical approval was obtained through both the NHS Health Research Authority (HRA) and the Research Ethics Committee at Lancaster University.

## Results

In total, 23 individuals took part in the study. Table 1 depicts the demographic characteristics of the participants.

**Table 1:** Demographic characteristics of study participants.

Characteristics		N (%)
Gender	Male	7 (32.5)
	Female	16 (67.5)
Age	30–40	2 (9.7)
	40–50	7 (31.3)
	≥50	14 (59)
Participant's role	Director of public health	1 (4.34)
	Link worker	6 (26.1)
	Service user	6 (26.1)
	Community provider	7 (30.43)
	NHS commissioner	2 (8.69)
	The Voluntary, Community, Faith and Social Enterprise Sector^2^ provider	1 (4.34)
Length of service	<10	8 (34.7)
	10–20	10 (44.2)
	≥20	5(21.1)

**Figure 1:**
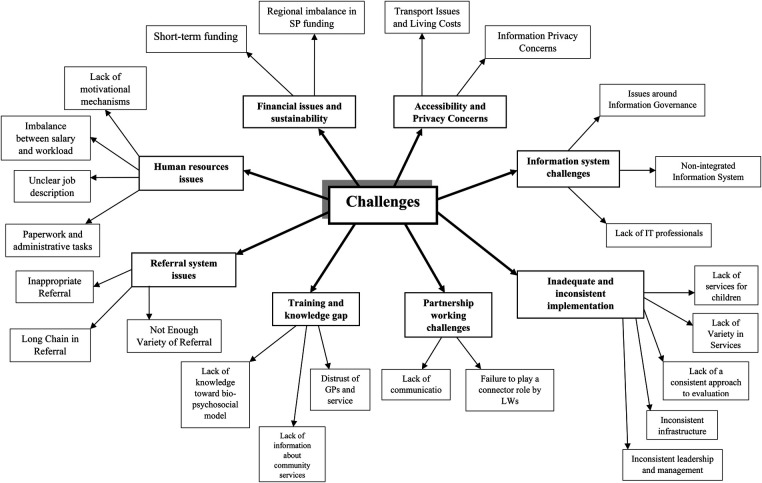
Map of themes and subthemes.

From the insights obtained through interviews and focus group discussions, we have identified eight themes from the perspective of the stakeholders. These were: financial issues and sustainability, human resources challenges, partnership working challenges, inadequate and inconsistent implementation, information system challenges, referral system issues, training and knowledge gaps, and accessibility and privacy concerns. The themes, and sub-themes are also depicted in Figure 1.

### Theme 1: financial issues and sustainability

Financial issues were important to all stakeholder groups; for health and social care professionals, this meant insufficient capacity to address a wide variety of needs. They believed that the lack of long-standing funding arrangements and inequality in the resources and relationship between NHS and the VCSE sector would result in significant concerns. *“They might be different things but the root cause is the same. It's money, it boils down to money” (NHS2)*.

#### 1-1: short-term funding

For CPs, short-term funding meant short-term contracts, lack of variety in service provision, and lack of permanency in location and offers. More specifically, they highlighted that short-term funding leads to unsustainable workforce contracts for those delivering the support. “*The most important support we want from the NHS is to provide job security for small organisations through securing long-term workforce contracts”* (CP2). Furthermore, CPs mentioned short-term funding as a significant barrier to the successful implementation of the SP programme particularly in response to a wide range of social and emotional needs of the population. *“Supporting people under emotional and psychological pressure, reducing loneliness for older people, educating children and young people to manage their emotions are among important actions of the programme”.* (CP4). They further explained that lack of funding can hinder the provision of permanent locations to offer community well-being services. “*It's really bad that we don't have a stable place to provide services and have to change our location now and then due to unstable financing”* (CP4).

They indicated that an injection of short-term funding to provide SP services in most of the catchment areas would impose high pressure on delivery partners to continue the sustainable provision of services. “Short-term funding causes uncertainty in the provision of different community wellbeing activities”. (CP5). They also declared their concern about small organisations continuing their funding in supporting local communities. “*Six-month or one-year service contract cannot suffice the provision of sustainable services”* (CP1). One of the CPs recommended a potential solution to give more control to the VCSE sector to shape the activities in line with the needs of the communities. “*What we do in Bay, which is a small local investment fund. So, we will offer applications, that are judged by a panel made up of citizens and other organisations and we were in contract with community organisations who can carry funds over from the financial year. This means that the activities are delivered, not through the NHS, the money goes out to our community providers, who can stretch it, deliver it, and tweak it how they want to”* (CP4).

One of the NHS participants clarified that Primary Care Networks (PCNs) could formally expand networks between the health sector and community support groups. However, some VCSE organisations were left without adequate financial support to provide the required services for those referred to them. “*Funders should consider the VCSE as an integrated part of the health and social care sector”* (NHS1).

Likewise, most of the EbEs were dissatisfied with the non-continuity of services. They asked for longer-term programmes and noted that 3 to 6 months contracts cannot meet their longer-term needs, and do not allow service providers to monitor improvement or deterioration in people's health status periodically. “*We need someone to constantly monitor our health, someone with enough time to check whether or not offered services were useful to us*” (EbE4).

LWs also mentioned unsustainable funding as an important challenge regarding the provision of long-lasting and quality SP services. “*Social prescribing initiatives have resulted in significant rises in referrals to community wellbeing activities that could not be sustainable unless providing additional funding for community-based organisations”. (LW4).* Most of the LWs complained about inadequate access to sustainable financial resources, noting that a significant portion of funds is allocated to LW salaries, yet they remain dissatisfied with their income. “*So, not enough resources, not enough financial incentives, and even not enough office space are among the problems we are always facing. Money affects our job*” (LW3).

#### 1-2: regional imbalance in SP funding

Most of the study participants agreed that some regions have a better financing function for SP initiatives. Consequently, uneven distribution of services within a region and an inadequate number of providers delivering social prescribing services in some geographical areas were among the important barriers. Some of the NHS staff explained that to improve disease prevention and develop more integrated models of health and social care, PCNs were constituted and financed by the NHS to provide additional funding for the primary care sector. They believed that insufficient collaboration between PCNs and some of the VCSE sector schemes particularly small organisations failed. *“This is because they want to go, they’re holding some funds, and they are not distributing it correctly, in the way that we think” (NHS3)*.

Likewise, some of the LWs reported regional funding imbalances for SP services. They believed that to secure social prescribing funding within PCNs, adequate finance should be given to link workers to help them support users with their variety of health needs. “*I think the logic behind allocating budget to different SP schemes should be the population size of an area, their demographics and socio-economic condition” (LW4)*.

### Theme 2: human resources issues

#### 2-1: lack of motivational mechanisms

Many NHS workers approve of the significant role of social prescribers in reducing the workload on GPs and healthcare professionals. However, they mentioned a lack of motivation among LWs and noted that a considerable number of these workers suffer from job burnout. “*How could we expect them to deliver adequate support services to the population when service providers are deprived of basic motivational mechanisms”?* (NHS4). Furthermore, the majority of study participants stated that a high turnover rate among LWs due to short-term employment contracts and low pay would result in limited human resources particularly those with sufficient knowledge, skills, and expertise. Accordingly, most of the CPs agreed that staffing shortages might end up with lost or delayed services for people with social needs. “*Some of the clients complain about long wait times when seeking care*” (CP2).

#### 2-2: imbalance between salary and workload

Some of the LWs highlighted that lack of balance between salary and workload acts as a barrier to a positive and productive work environment. *“When there is no balance between salary and workload, there won't be any desire to continue serving as a link worker”. (LW2).* Several link workers added that they often face pressure due to an overwhelming number of referrals, largely stemming from the unclear understanding of social prescribing and its referral system to GPs. “*Some of them ring us almost every day and make us deliver services more than our remit” (LW3).* Although they confirmed that social prescribing is for everyone, they acknowledged their limited capacity to provide services and resource deficiencies that put pressure on the system. *“We face with many difficulties to meet the needs of a wider group of people being referred to us?” (LW1)*.

#### 2-3: unclear job description

Two NHS commissioners agreed that although there is a unified job description for LWs in the UK, SP service delivery is quite different in most of the regions. They explained that some of the LWs just limit themselves to signpost users to relevant local resources; while others play more active roles and provide holistic services to improve health and wellbeing outcomes for people. “*I see many link workers spend enough time networking, engaging in existing activities, and increasing referral pathways to include as many vulnerable populations as they can; however, there are still some link workers who do not know their special position in providing non-clinical services” (NHS2).* These inconsistencies were mentioned as key barriers stemming from the different professional backgrounds of link workers*. “Normally, people who work as link workers come from a broad range of professional backgrounds and face with lack of role clarity.* (NHS6). A link worker also added: *“I signed someone off because, after I’d spoken to them, they said about suicidal stuff, I was told by NHS, “That's not your role, boom, wash your hands, signpost them off quickly, you are not a mental worker”. (LW3)*.

#### 2-4: paperwork and administrative tasks

Most of the LWs agreed that spending too much time on paperwork and administrative tasks inhibited them from fulfilling their main responsibilities. Likewise, LWs discussed that having no access to a clear job description and task prioritising guideline led them to concentrate more on tick boxing and admin duties, which potentially are mentioned as important factors for taking them away from service users. “*Most of the time I'm busy with doing admin work rather than spending time with users"? (LW4)*.

### Theme 3: partnership working challenges

#### 3-1: lack of communication

Lack of understanding of mutual expectations, and desirable outcomes between different partners of the SP programme was one of the main obstacles that was emphasized by most of the respondents.

“*What I'm learning is that the ICB is all about the NHS; they don't seem to have as much political control over the GP Fed and the PCNs because GP practices are private businesses. They're in the NHS but they're kind of not joining up and they're not particularly good at joining in”* (NHS6). One of the CPs delved deeper into the matter of cultural differences that undermine effective communication across sectors. Accordingly, she shared her negative experience from one of the recent meetings that have been held with NHS people, service providers, and representatives of the council to discuss improving the physical and mental health outcomes and wellbeing of people.

“*About five minutes to go before the end of the meeting, I started to talk about measurements and how we were going to do that and I was making the point and so on. The PCNs had all started to argue between themselves, one had got its issues, comorbidity concerns, or whatever they call them were; I don't know, mental health. I said Oh my Gosh, why don't they understand what I'm talking about”* (CP2). The community provider continued that the priorities of different sectors are not properly aligned. “*Well, we're something else. I was like, for God's sake, it's not about that, it's a little system, easy stuff, let me explain more. But time ran out and the meeting ended without any consensus on the concept”* (CP2). Another participant noted that PCNs do not effectively work with the VCSE sector which will ultimately lose their effective connections. “*I believe VCSEs are not engaged early enough in designing appropriate mechanisms for delivery and funding processes”* (CP4).

A group of participants believed that the lack of a systemic approach and attitude in the government toward the critical role of CPs in promoting the population's health status creates a significant challenge. “*But I do not think we will see sustainable recurrent funding in the way that we do to our statutory services until we have that kind of change to the mindset of individuals at the government level*” (CP3).

#### 3-2: failure to play a connector role by LWs

LWs called themselves connectors, having the ability and skills to develop constructive relationships with other partners of the programme. “*If I recognise that different options are available in the VCSE, I can easily assess people's needs and refer them to suitable services. This kind of information is better obtained through periodic gatherings and group meetings” (LW3).* However, some of the LWs complained about not being informed of networking events. “*Most of the time, we are not informed of regular group meetings, so there is no opportunity for us to share our experiences with others “(LW1).* Some of the LWs highlighted their role of giving feedback on service users’ journey to GPs as a facilitator to help them understand how SP services are effective. However, the lack of sufficient infrastructure to establish such interactions was considered an important barrier. *“Regular feedback is crucial for delivering high-quality services, but most of the time GPs do not have time to discuss about different cases and their main issues” (LW4)*.

### Theme 4: inadequate and inconsistent implementation

#### 4-1: inconsistent leadership and management

One of the main challenges that most of the attendees agreed on was improper management and lack of supervision of SP implementation processes in some of the regions. “*Link workers should closely work with their line managers to receive consistent supervision to empower them to cope successfully with difficult issues”. (NHS2).* They added that the lack of proper supervising mechanisms to allow SP services to be delivered based on quality indicators and person-centred approaches is of great importance. *“What are the training and development needs? How they should be met? Who should provide regular supervision of SP services? Who should develop a cooperative scheme? Who should organize collaborative teams including the local community, and service providers in a way to ensure their uniformity and the provision of coordinated support? These are some of the main questions that arise to highlight the importance of good supervision in place”. (NHS1)*

#### 4-2: inconsistent infrastructure

Most of the study participants mentioned inconsistent infrastructures in different geographical regions as the main barrier to equal service delivery. “*In some GP surgeries there is even no office space for link workers. We are aware of such limitations but an inclusive and ongoing cooperation is needed to resolve these issues” (NHS6).* Another participant added “*It's a shame that some vulnerable people don't have appropriate access to SP services, and some others are lucky in their area” (NHS3).* Physical facilities such as office space, computer devices, telephones, and other working equipment have been reported by link workers as barriers to enhancing service delivery among the population. “*It's absolutely clear that we need to be provided with* adequate space and physical *resources* to accommodate our specific needs properly”. (LW4).

#### 4-3: lack of services for children

Study participants confirmed that lack of attention to the psychological and social needs of children and adolescents from the early years of their lives is one of the important shortcomings of the SP programme. “*Social prescribing should start from schools, even nurseries; we need to know young people, how to work with them, and how to meet their special needs”.* (NHS4)

#### 4-4: lack of consistent approach to programme evaluation

Some of the LWs explained that a lack of a cohesive approach to SP programme evaluation would lead to a delayed and disordered delivery of services. “*Lack of clear standards is a big challenge; it stops the system from running an objective evaluation on what is working well and what can work better”* (LW5). They further explained that service users could act as the most important source of information to know the quality and quantity of implemented procedures and delivered services. “*We need individuals’ perspectives to better understand what happened during the delivery of SP services, what is important to users, and most importantly, how to improve the SP system through the use of this data”* (LW3). Short-term nature of the evaluation approaches was another issue acknowledged by some of the LWs. “*Also, I look at the long-term funding, as you said. You can evaluate it much better over years rather than just a year or two”* (LW5).

#### 4-5: lack of variety in services

Some of the NHS staff explained that a different range of key needs should be taken into account when designing SP services. “*These needs might be due to emotional and mental health problems, social isolation, and financial issues” (NHS3).* Based on the study participants’ viewpoint the endpoint for all service users is to be heard and cared for patiently without any rush *“This won't be achieved unless we prepare them tailored-based SP services”* (NHS4). They also added that people's health and well-being condition is mainly affected by their socio-economic condition. Consequently, they mentioned SP as one of the most effective mechanisms to meet the holistic needs of service users. *“There is no escaping the fact that all of us have different levels of social, emotional, mental, and even financial needs that need to be satisfied”* (NHS 4). Concerning the importance of tailored-based services, some of the service users believed that, a wider set of services should be provided at the community level to meet their needs more effectively. “*I think there is still a need to provide more services in the fields of sports, music, and art” (EbE4).* They highlighted that a limited scope of services cannot meet the diverse needs of people and ultimately hinders the effectiveness of services. “*Our voice needs to be heard; we must have control over what is offered to us” (EbE1).* Experts by experience also discussed the challenge of being supported by cases of suffering from severe mental health problems. “*When you are referred to a community service with complicated mental health issues, it's evident that lots of these problems are immensely tied into your social condition” (EbE1)*.

### Theme 5: information system challenges

#### 5-1: issues around information governance

Most of the study participants believed that the lack of an integrated information system for SP is a serious challenge. “I strongly believe that each member of the SP team wants to have access to the required information in the fastest possible way” (NHS2). One of the NHS staff also emphasized the necessity of knowing about available services in the community and the impact of SP services on different partners of the programme. “We need such transparent data to address social determinants of health and build trust for a long time” (NHS3). Accordingly, one of the CPs stated that the lack of a right linkage between the Emergency Medicine Information System (EMIS) and Primary Care System (PCS) directory hinders a successful connection of care across different healthcare settings. “To successfully provide support services for the population, we need to access necessary information about their demographics, medical history, and other health information” (CP1).

#### 5-2: Non-integrated information system

Most of the NHS staff affirmed that although digital technologies in SP facilitated the process of getting relevant information on individual users, it is still difficult to navigate housing, health, benefits, and legal issues of the population. Accordingly, one of them highlighted the key role of data on social determinants of health to support LWs’ work. “*Digital SP is not just about online monitoring of people in need or providing them 24-hour, 7 days a week connection. LWs need to be informed of the key role of social factors like housing, education, income, and a range of environmental factors” (NHS2).* Some of the LWs stated that lack of access to clinical leading systems across different GP surgeries would act as a barrier to performing adhesively. “*I wish we could get access to clinical leading systems such as EMIS to provide us additional support and relieve some burden on us” (LW5)*.

Another barrier was the lack of up-to-date information about available services in the area and often relying on word of mouth from members of the community. CPs suggested that a straightforward and user-friendly Community and Voluntary Solutions (CVS) directory could build participatory networks to provide practical assistance around funding, volunteering, and referral processes. “*We need to be trained on how to work with CVS, how to extract needed data, and how to link the population with a variety of SP services in the area*” (CP4).

#### 5-3: lack of It professionals

Technical difficulties and lack of information technology (IT) professionals were other key cited challenges by NHS participants. Indeed, they believed that to properly respond to technical issues, there is a necessity for recruiting trained information technologists to use information systems, or platforms flawlessly. They added that allocating a sufficient number of IT professionals to use a digital-based technology is necessary to obtain relevant information on the personal health profiles of the population and consequently offer tailored-based services. “*We lack IT staff; we cannot ensure that link workers offer person-centered services”* (NHS5).

### Theme 6: referral system issues

#### 6-1: long chain in referral

The long waiting list was a challenge that was mentioned by CPs as an important factor in making the referral system slow and difficult for users to understand. “*Waiting lists keep growing because the need for mental health services is increasing every day”* (CP3). Accordingly, EbEs declared their dissatisfaction with long waiting times to get access to SP services. “*And somebody that is in a crisis and needing some service, you know, six months is a very long time, they can deteriorate in that period*” (EE6). Most of them added that there's an expectation of a short waiting list, particularly for people with urgent needs. “*We were just worried about receiving timely support without any hesitation*”. (EE2). Some of the CPs further noted that a lack of follow-up services after the initial referral of individuals to social prescribing would hinder people from actively engaging in their health condition. “*To encourage people to take an active role in realising their health, on-time feedback and support are needed”* (CP2).

#### 6-2: not enough variety of referral options

The lack of various referral options was another challenge that was mentioned by some of the LWs. “*So, making sure that the referral is appropriate for a person, demands a variety of services available at the community” (LW6).* On the other hand, CPs explained that link workers are not only supposed to limit their tasks to signposting individuals to relevant community assets but also, they are expected to actively communicate with the population, identify people's needs, and provide them suitable options. “*With help from link workers, we can create more sustainable support*” (CP3). Attendees added that an important barrier in referring people to related and need-based activities is the lack of local appropriate services in some geographical areas. “*With no doubt, I should say that lack of community assets would cause a significant gap in services*” (CP3).

#### 6-3: inappropriate referral system

When discussing the referral system, the majority of link workers highlighted various challenges, including high GP workloads that leave them with inadequate time for making appropriate referrals, as well as unclear standards to objectively determine who should be referred to whom. “*GPs should properly decide who is suitable for the referral. How can this decision be made correctly unless enough information is given to them regarding the existing indicators and guidelines”. (LW2).* LWs further explained that the right referral requires the right decision and to do so, a good connector is needed to bridge service users with local support activities. “*I agree that GPs face with lack of time while delivering services; but if they accept us as their supporting forces, we can spend more time with people who struggle to manage their healthcare” (LW1)*.

### Theme 7: training and knowledge gaps

#### 7-1: lack of knowledge toward bio-psychosocial model of health

Some of the NHS workers believed that promoting health is not possible unless adequate attention is given to basic health conditions such as proper housing, education, employment, income, and a supportable ecosystem. “*It is impossible to reach the highest level of health without having self-control over social determinants of health”.* (NHS2). Some others further explained that increasing demand for receiving consultation services for non-clinical health issues is a significant element of the biopsychosocial model of health in which a variety of physical, psychological, and social factors play a significant role in the population's health condition. “*Sometimes just by providing befriending and social networking opportunities for the population, we can avoid isolation, loneliness, and depressive symptoms. (NHS3).* Despite the undeniable importance of this model, health professionals might dismiss the benefits of SDH as inseparable parts of the care process. *“Instead, social prescribing services can provide GPs an alternative to consider underlying factors contributing to health issues” (NHS1)*.

#### 7-2: distrust of GPs and service users towards social services

A group of study participants believed that a lack of awareness about SP services and their potential advantages, particularly among GPs and service users, would result in the unfitting reception of services. One of the CPs stated that: “*Still many people look at clinical services as superior services that work miracles in people's recovery”* (CP2). They introduced GP surgeries as suitable places to advertise SP services and promote people's knowledge regarding its standard pathway. “*Whereas if we do a video of our dance activity, it's a relevant and accessible thing, it can be screened in doctor's surgeries that other people can see on Instagram and blah, blah, blah, and speaks to that person who would have otherwise gone to the doctor and said, “I’m sick, I need this.”* (CP4).

Similarly, most EbEs considered their lack of understanding of the SP system and its potential advantages as a main obstacle that prevents the programme from being widely accepted. “*This is the first time that we got acquainted with the title of social prescribing in this meeting and we realised that the services provided to us in community centers were actually in the form of social prescribing”* (EbE3). They added that transferring information through the use of posters, leaflets, or other formats of teaser advertisements can potentially boost people's knowledge about the effectiveness of non-clinical solutions including SP services and community well-being activities. “*In GP surgeries it takes some time to enter the GP's office; that time is a good opportunity to watch a short video about social prescribing in waiting areas”.* (EbE1).

Limited understanding of the SP pathway was another concern raised by some of the NHS staff. “*We need to increase service users’ awareness, and then use their capacity to link with the voluntary, community, and social enterprise organizations”* (NHS2). They declared that most users access SP services without being aware of its implementation process, and underlying mechanisms. “*Although people are key resources for providing social prescribing activities, this support is often underused”* (NHS5). They further explained that a low level of community engagement is a crucial barrier to the successful implementation of SP. *“If people do not trust in link workers, community providers, and the entire system they will never succeed in addressing their problems” (NHS1).* Some of the service users also mentioned that a lack of appropriate knowledge about SP services among clinicians and those working in primary care networks would hinder the effective transfer of information to service users. “*Well, my husband found out about a well-being group in Cornforth; he saw a poster in the doctor's surgery in Cornforth and he asked the receptionist about it and they didn't know anything about it”* (EbE2).

Some of them also believed that a lack of trust in non-clinical solutions inevitably worsens health outcomes and leads to decreased compliance with improving lifestyle strategies. “*Most of us consider physicians as warriors and life saviors, so if they promote SP services, it will have a better effect”* (EbE2). They further explained that the reason behind the dominant culture of medicalization is the distrust of GPs towards social services and their benefits. “*GPs put us on tablets but when we are spoken to, it turns out that what happened is that we've had an abusive relationship, we're downtrodden, and what we want is some abuse counseling rather than happy pills” (EbE1)*.

#### 7-3: lack of information about community services

Some of the LWs mentioned that inadequate training to prepare them for developing trusting relationships with service users and fulfilling their psychological and social needs in the best possible manner was an important barrier. They noted that as the landscape of local assets continuously changes, it is of great importance to receive sufficient and up-to-date information about local community services regularly. Indeed, transparent information about available services in the community could act as a key strategy to increase LWs’ understanding of what is available and how they can make the best use of support activities promptly. “*We need to learn more about available services in the community and different coping strategies for service users.”* (LW3). Some LWs also explained that they need to be equipped with a variety of skills ranging from counseling, crisis management, emotional resilience, proper IT skills, and advocacy to support service users across their journey into social prescribing. “*We are overwhelmed with a variety of roles and responsibilities while there are some boundaries on our knowledge and skills”.* (LW4).

Being trained especially in mental health, first aid, safeguarding, life skills, confidentiality, and respect for people's needs was also mentioned by CPs as a key determinant for significantly increasing the quality of services provided by them. “*In the field of mental health, specific skills are necessary for staff to be aware of people's background, and condition and possible ways to provide them proper services”* (CP4). They further explained that small-group discussions act as a powerful learning strategy that enables effective communication among learners and promotes their literacy around different non-clinical health topics. They added that under time constraints, it is possible to use a variety of learning methods including online classes to achieve the desired educational goals. “*Virtual* learning is, in fact, a*n efficient platform for swift and effective training of staff who look for maximum accessibility, flexibility, and time-saving”* (CP2).

### Theme 8: accessibility and privacy concerns

#### 8-1: transport issues and living costs

Transport issues to the prescribed services were identified as an important barrier to accessing SP services, particularly in geographic areas that are outside towns and cities. “*I don't drive, so it is much harder for me. I have to walk, get the bus, or sometimes take a taxi which causes me lots of cost and trouble”* (EbE2). EbEs also felt that poverty made it much harder to achieve their health goals. They explained that when people face difficulty in meeting their basic needs and living expenses it would be more difficult for them to seek help to address their psychological and social needs. “*I think the other challenge is a cost-of-living crisis. I’m worried about where my rent is coming from, it is also societal”* (EbE2). One of the LWs gave an example:

“*I can mention one of my clients who is seventy-five years old and needs others’ help to attend a healthcare center; she always complains about transportation costs as her house is further away from the center. We cannot go and* *bring her to the center because we don't have the required staff and facilities; this is a barrier”* (LW6). One of the CPs acknowledged that population groups that are more vulnerable and exposed to greater social and economic disadvantages, as well as those with disabilities and chronic conditions face more challenges regarding transportation. “*We need some supportive programs for low-income individuals,* *minority racial groups, and persons with disabilities”* (CP1).

#### 8-2: information privacy concerns

Some of the SUs mentioned their mental health condition as sensitive personal information and expressed concerns about its disclosure while getting in touch with community well-being activities. “*I’m not sure whether they will keep my personal information secure or not”.* (EE1).

#### 8-3: anxiety and agoraphobia

Most of the service users mentioned that suffering from anxiety disorders as well as group phobia made it hard for them to leave their houses and seek help. They also expressed their discomfort in joining groups and mentioned a lack of self-confidence as the main contributing factor. “*At the beginning, I was scared to talk to people and communicate with them properly”* (EbE4). The role of link workers in building self-confidence and independence among service users was also highlighted by the study participants. They added that education on ways to improve their self-reliance and actively engage with SP services is necessary to help them mitigate their loneliness and mental health issues. “*We need someone to help us select and go through appropriate socially prescribed activities”* (EbE1).

## Discussion

There is a growing interest in engaging stakeholders in research to ensure that their knowledge, expertise, and perspectives are included, thus increasing the applicability of research findings. One of the important contributions of our study is the development of comprehensive ideas based on different stakeholders’ needs and expectations, aimed at recognizing possible obstacles to success. The findings and their implications are discussed thematically below. The identified barriers to successful SP implementation included financial matters, lack of collaboration, inconsistency of SP delivery, lack of knowledge, resources, training, appropriate evaluation systems, integrated information system, issues in human resources, referral system, supervision and leadership, and the lack of a holistic approach toward health and failure to consider the bio-psychosocial model (11, 17–23).

### Financial issues

In line with most previous studies, the lack of long-term funding was a significant challenge, which was mainly related to the SP programme's reliance on the governmental budget (24). Literature affirmed that in most cases, grants are allocated to third parties by local authorities. Therefore, the programme will adversely be affected by budget deficits and the overall economic condition of the society and the local authority (25). Accordingly, the study participants declared that NHS commissioners, funders, local authorities, service providers, and community members have a key role in ensuring the sustainability of social prescribing. Thus, a lack of collaboration between different partners of the programme could lead to several problems, including missed opportunities for engaging in volunteering activities and support networks (24). Likewise, in another study, a group of respondents stated that lack of local assets and inadequate community engagement both hindered the project from being sufficient and sustainable (19).

### Partnership working challenges

Improper communication and ineffective relationships between different partners of the programme were also mentioned as significant barriers to SP success. Different functions of the programme, including financing, the provision of physical facilities, manpower resources, and information sharing, are influenced by the way different stakeholders are engaged in a collaboration process. Lack of knowledge about team meetings and social events that are routinely held with the presence of SP partners was also a barrier to enabling the sharing of ideas and gaining the benefits of collaboration across sectors (20). Most of the study participants highlighted local forums and regular catch-up meetings as important platforms for bringing organisations together to address problems collectively. They further noted that differences in language and understanding between NHS and VCSE organisations may lead to different priorities, particularly regarding determinants of health (21). Factors such as inconsistency of the programme, lack of trust, conflicting goals, lack of clear decision-making processes, and information deficits were among the factors that negatively influenced collaboration across SP stakeholders. Similar findings highlighted that failure to set priority for addressing the health needs of vulnerable and isolated groups, and short-term relationships between different stakeholders of the programme, could lead to a barrier to effective communication and programme development. Participants mentioned that these undesirable features in the workplace might originate from improper and unsupportive management (22, 23).

Based on the interviewees’ viewpoint, one of the barriers to effective interactions is the lack of a common goal between different partners. This shared vision can be developed by committed leadership within PCNs and Integrated Care Systems to develop collaborative processes with local communities and make a broad understanding of social prescribing vision throughout the system (26). Inadequate funding for collaborative projects was another barrier to developing the role of the VCSE sector and its contribution to the health outcomes of the population. Accordingly, similar literature confirmed that users are moved back and forth across different sectors considering their varying needs (27). Therefore, long-term coordination between various settings and sectors is of great importance. Previous studies have also highlighted the importance of collaborative relationships between PCNs, link workers, and community providers (28, 29). Findings also revealed that such a collaboration will grow into a landscape where NHS commissioners and community providers reorganise themselves into integrated care systems (ICSs). Such a system will also ensure that ICSs provide sufficient resources for community providers to act as part of co-producing engagement plans (28, 30).

### Information system challenges

Another challenge regarding digital supporting social prescribing was its failure to evaluate outcomes and provide potential measurement of progress along the SP journey. Study participants believed that the benefits of digital social prescribing are much wider than a simple database or an online directory of services. They added that working as a referral management platform will support its users to take advantage of available SP services. Likewise, its potential to provide an effective evaluation system with integration into key information systems like EMIS was mentioned as an important supporting area for organisations that deliver services within communities (31). Several studies affirmed the significant role of SP platforms to provide an updated online directory of services, including information on community members’ medical history, demographics, and socio-economic characteristics, and most importantly manage referrals through analysing the system's capacity. Lack of integrity between different information systems in the health sector including GP clinical systems was also a crucial barrier to support different sectors in delivering SP services in an effective, holistic way (32). Study participants also acknowledged that connecting different systems and their databases is one of the main challenges that require interoperability for those engaged in social prescribing activities and the digital maturity of the system.

The lack of an integrated digital platform within the NHS was an important obstacle mentioned for monitoring people's referral status and their progress throughout the SP journey (33). Such a deficit was supposed to hinder GPs from being actively involved in the referral system, regularly tracking individuals during their health journey, analysing the impact of SP on health outcomes, and automatically updating patients’ records across different information systems (31–33).

### Referral system issues

The high volume of referrals and long waiting lists were also mentioned as significant barriers which were supposed to be related to a lack of shared understanding between commissioners, providers, service users, and partners from different sectors (28, 29). Evidence confirmed that lack of shared understanding and trust in different partners due to inadequate training and low levels of confidence to recognize the significant role of social determinants were other key barriers to making effective referrals (28, 30). Some of the literature revealed that to facilitate referral processes, it is necessary to develop and apply standard guidelines and unified criteria to ensure referral integrity across different settings (28, 33). Department of Health's national evaluation agreed with the findings and added that inadequate local infrastructure is a challenging factor for the delivery of SP interventions. A lack of facilities and physical resources will restrict available services in the community so that they are not responsive to the varied needs of the population and cannot meet their expectations properly (34).

### Inadequate and inconsistent implementation

As most of the SP services include community providers in the VCSE sector, the lack of local infrastructure and physical resources was mentioned as another key obstacle to delivering inclusive and high-quality services to users. Inappropriate access to basic facilities hinders the socio-economic development of a society and makes some geographical areas isolated due to the absence of physical or institutional infrastructure (22). The provision of suitable office space for link workers alongside required facilities, computer devices, telephone access, standard guidelines, and safety procedures are among the important necessities for the successful implementation of the programme. In line with several findings, infrastructure investment imposes a serious burden on the government. Thus, applying appropriate incentive mechanisms to take advantage of private funders can play an effective role in tackling the problem of poor infrastructure and meeting time and budget requirements for service delivery (35).

Furthermore, having access to standard procedures in different areas was a key solution against inconsistency in service provision, and the referral system (36). Moreover, the inability to deal with complex mental health-related issues alongside diverse backgrounds of people who refer to healthcare settings was a significant challenge in the workplace and most of the providers agreed on its prominence (37).

Lack of an appropriate evaluation system and improper management were also a crucial challenge that most of the study participants emphasized on its importance. The investment of financial resources in paying salaries to link workers and neglecting the importance of proper management through the use of precise and clearly defined evaluation indicators and systematic evaluation procedures were among other important challenges mentioned by the participants (38).

### Human resources challenges

As a human resource issue, frustration among link workers and other social care providers was mentioned as a critical challenge, particularly in the areas where the load of referrals is very high and inadequate salary and infrastructure assets put significant pressure on providers (36, 37). Furthermore, the lack of well-being support activities for employees and irregular supervision of their services were among other important barriers to ensuring both peer support and information sharing to discuss existing challenges (39).

### Lack of supportive knowledge and attitude

Failure to adopt a holistic approach toward health and irresponsibility to consider the bio-psychosocial model instead of a mere reliance on biomedical and clinical factors were also agreed by the majority of participants as important barriers. The bio-psychosocial model focuses on a more individualistic approach to patient care and considers different emotions, intellectual factors, and social needs of people based on their unique conditions (40). Accordingly, social prescribing is mentioned as an inclusive person-centered approach to health and well-being that includes a wide range of activities from signposting to proper services to employment or financial advice, and more significantly offering sustainable support to those suffering from loneliness or mental health issues (40). This approach led to an important shift from the biomedical model to the biopsychosocial model of healthcare focusing on social determinants of care to help people manage their chronic condition, address health inequalities, and build effective social support networks (41). Link workers believed that the lack of training for service users and GPs about the potential of SP in the improvement of population health conditions was an influencing factor in increasing the burden on link workers and inappropriate referrals (26). Some others acknowledged the lack of incentive mechanisms for service providers as a barrier to engaging them in the programme with their utmost capacity (42).

### Strengths and limitations of the study

To the best of the authors’ knowledge, there is no research bringing different stakeholders’ viewpoints together to determine the challenges in the implementation of SP. By benefiting from others’ knowledge, it would be possible to build consensus via a collaborative approach to identify potential challenges and opportunities. In a definition by Concannon et al., the 7Ps Framework was developed to identify key stakeholders who should be considered for engagement. They mainly included patients and service users, providers, payers, policy-makers, and policy advocates whose participation would increase the transparency and relevance of the project, and consequently increase its compliance with best practices (14). In the case of SP, stakeholder engagement facilitates community development and an efficient use of local assets as well as allowing service users to form their peer support network in achieving positive sustainability steps (12). To achieve this purpose, the views of four stakeholder groups including service users, community providers, link workers, and NHS staff who were dealing with coordinating or leading the programme were incorporated into the current study. A combination of qualitative methods, including interviews and focus group discussions, was used to involve multiple stakeholders in identifying existing challenges to improve the SP system. Furthermore, thick description was used to describe the context in depth, which will aid the transferability of the findings to similar contexts.

However, there are a number of limitations in this study. The study was conducted in Lancashire and South Cumbria, areas known for high levels of deprivation. Consequently, findings may not be fully transferable to other regions with different socio-economic or healthcare landscapes. Furthermore, while the study included a diverse range of stakeholders, it did not capture all perspectives equally. For example, the views of policy-makers and funders were underrepresented, which might limit the understanding of broader systemic challenges. Third, the study does not provide long-term insights into the impact of identified barriers on SP outcomes over time. A longitudinal approach could offer a more comprehensive view of the sustainability and evolution of the implementation challenges. Finally, the study did not assess the effectiveness of proposed solutions to the identified barriers, which limits its applicability in guiding specific interventions. Future research should include pilot testing of strategies aimed at addressing the identified challenges.

## Conclusion

Developing an evidence base on barriers to the successful implementation of SP allows policymakers and service providers to overcome potential challenges and improve the implementation process of SP services. Findings will also help us improve the SP programme on what challenges exist for whom, and what feasible strategies can be developed to tackle implementation issues. Furthermore, it would be beneficial for planners, policymakers, and providers to gain a comprehensive understanding of the SP programme and existing challenges. Findings revealed that most of the challenges are rooted in funding issues, inconsistent management, lack of proper communication between different partners of the programme, failure to consider a wider SDH, ineffective information system, lack of an appropriate evaluation system with clear indicators for assessing quality services, and lack of knowledge and awareness. Using participatory methods provided us a comprehensive insight from different stakeholders to identify the main challenges in the way of successful implementation of the SP programme. This approach may help policymakers develop realistic policies to support social care within Integrated Care Systems.

## Data Availability

The original contributions presented in the study are included in the article/Supplementary Material, further inquiries can be directed to the corresponding author.

## Footnotes

^1^Anyone who has accessed (or is eligible to access) community-wellbeing activities and social prescribing services.

^2^VCFSE
