# Supplementary material for: Stakeholder views on addressing challenges to the implementation of social prescribing in the United Kingdom
Source: Front Health Serv. 2024 Oct 11;4:1413711. doi: 10.3389/frhs.2024.1413711 (PMC11502469; doi:10.3389/frhs.2024.1413711)
Supplement: Supplementary file 1 [file Table1.docx]

Table 1. Questions used for Focus Group Discussions

| **Interviewee** | **Main discussion points** | **Detailed questions** |
| --- | --- | --- |
| Community providers | Just think about social prescribing in nature (its mission, goal, objectives, and values) | Do you think social prescribing is a good idea?  -How do you see the program? |
|  | Now, think about social prescribing system and its referral process | -How does it work in your opinion?  -How effective and efficient is the system? |
|  | In case you have chosen somehow or completely ineffective and inefficient, please mention important challenges, and barriers regarding social prescribing system/ or the process of offering service to users. | -please mention any challenges come to your mind in any related category including financial matters, human resources, physical resources, referral system, information system, knowledge and awareness, available services, and connection between different partners of the program. |
|  | Now consider your mentioned challenges; select the most important one in each category, and ask yourself why such challenges exist? | Just pay attention, you should ask why for 5 times successively because we want to reach the root cause of each existing challenge. |
|  | Finally, please think of a practical recommendation/ solution or improvement strategy for each of the mentioned challenges based on the identified root causes in the previous activity. | - |
| Experts by experience/ or Service users | Just think about social prescribing in nature (its goal, and objectives); | -Do you think social prescribing is a good idea?  -Is it a helpful programme that can meet your social and mental health needs? |
|  | please think about the time that received social prescribing services or community wellbeing activities; | -What was your expectation from the programme?  -What did you actually receive from the program?  -Did it fully fulfil your expectations and need? |
|  | Please go back to your experience regarding social prescribing services. | -Did it make any change in your life?  -Please mention one of the best experiences that you had during your social prescribing journey.  -Please mention one of the most important challenges that you faced during this journey.  -How did you tackle the problem/ challenge? |
|  | Now consider social prescribing referral system; | -What were the most important challenges that you face with?  -Please think about any challenges you faced and try to put them in below categories.  -Also, if you think the challenges you want to mention cannot be categorized in any of the below groups, please just mention them.  (availability of services, accessibility of services, Decision making for choosing a suitable service, waiting time, connection with link workers, Connection with service providers, Relevancy of the service to your needs, continuity of services, Encouraging/ incentive mechanisms). |
|  | Please think about your connection with link workers and community providers. | -Do you see the connection to be collaborative, and participative?  -Do they listen to your needs and receive your feedbacks regularly?  -Have they ever changed any specific service based on your feedbacks and requests? |
|  | Finally, please go back to identified challenges. | -How do you think these challenges can be resolved?  -Please think about some practical solutions for each of the mentioned challenges. |
| Link workers and NHS commissioners | Just think about social prescribing in nature (its mission, goal, objectives, and values); | -Do you think social prescribing is a good idea?  -How do you see the program? |
|  | Please think about your job-related roles. | -Can you mention some of your most important job roles? |
|  | Please think about social prescribing referral system. | -How do you see this system?  -Is it straight forward and easy to navigate?  -Is it effective and efficient? |
|  | In case you have chosen somehow or completely ineffective and inefficient, please mention important challenges, and barriers regarding social prescribing system/ or the process of offering service to users. | Provide each of the challenges in the related category mentioned below.  (financial matters, human resources, physical resources, referral system, information system, knowledge and awareness, available services, and connection between different partners of the program). |
|  | Now consider your mentioned challenges; select the most important one in each category, and ask yourself why such challenges exist? | Just pay attention, you should ask why for 5 times successively because we want to reach the root cause of each existing challenge. |
|  | Please think of a practical recommendation/ solution or improvement strategy for each of the mentioned challenges based on the identified root causes in the previous activity. | - |
