# Supplementary figures and images for: Stakeholder views on addressing challenges to the implementation of social prescribing in the United Kingdom
Source: Front Health Serv. 2024 Oct 11;4:1413711. doi: 10.3389/frhs.2024.1413711 (PMC11502469; doi:10.3389/frhs.2024.1413711)

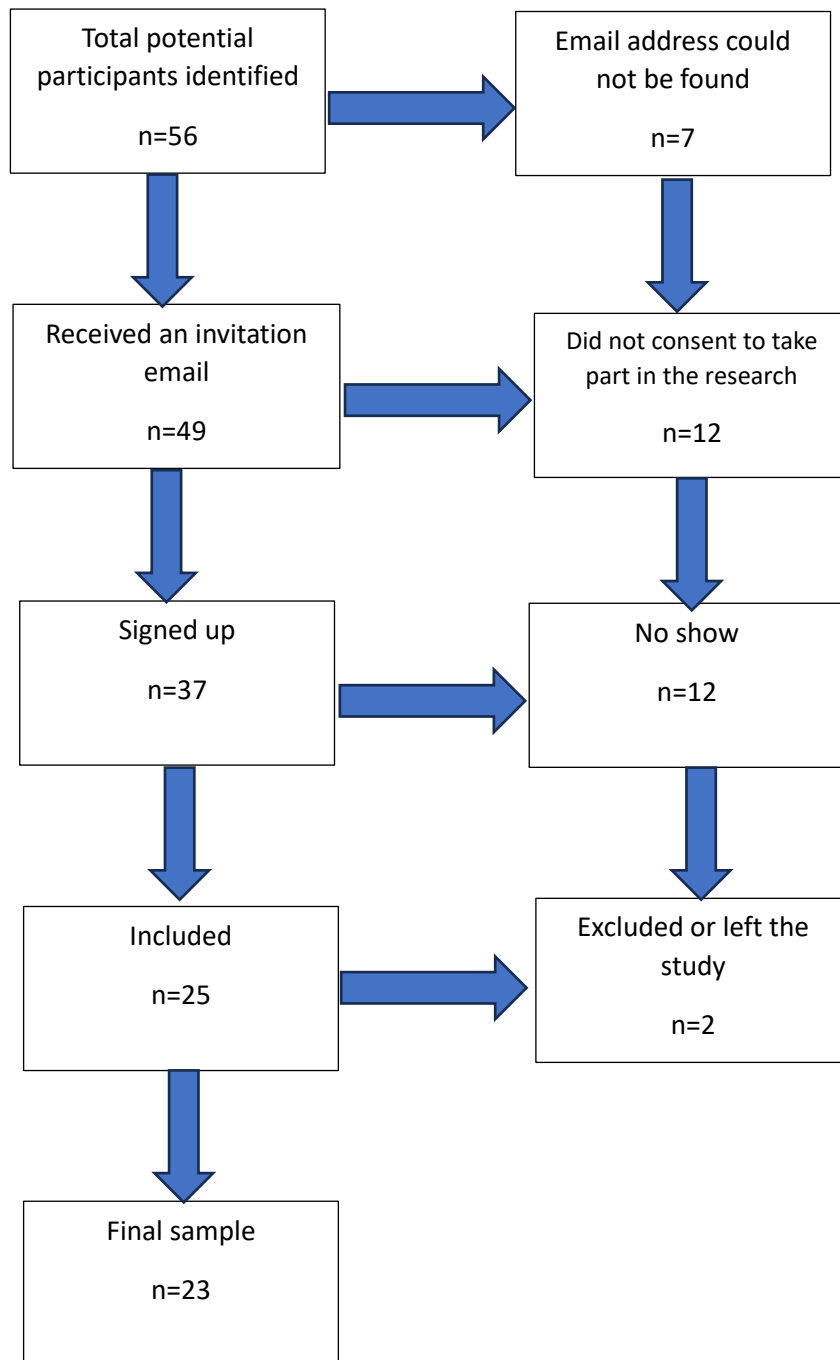

Recruitment process flowchart

Supplement: Supplementary file 2 [file Datasheet1.pdf]
